# Editorial: Biological Engagement Programs: Reducing Threats and Strengthening Global Health Security Through Scientific Collaboration

**DOI:** 10.3389/fpubh.2017.00148

**Published:** 2017-07-12

**Authors:** Jeanne M. Fair

**Affiliations:** ^1^Biosecurity and Public Health, Los Alamos National Laboratory (DOE), Los Alamos, NM, United States

**Keywords:** cooperative engagement, emerging diseases, biothreat, Global Health Security Agenda, One Health

**The Editorial on the Research Topic**

**Biological Engagement Programs: Reducing Threats and Strengthening Global Health Security Through Scientific Collaboration**

It is often said about infectious diseases that a “threat anywhere is a threat everywhere,” and the recent outbreaks of Ebola in West Africa and Zika virus in South America have proven that pathogens know no borders. Not only are they transboundary, pathogens do not discriminate who they infect. In addition to the natural increase in emerging zoonotic infectious diseases worldwide due to changing environmental conditions and globalization, the use of infectious diseases as warfare agents is a threat in today’s world. Early detection remains one of the best ways to prevent small outbreaks becoming epidemics and pandemics. Accurate diagnosis, detection, and reporting of diseases are important components of mitigating outbreaks, and biosurveillance remains the top tool in our toolbox. While vaccines have been important for controlling more common infectious virus diseases, they are less feasible for less common diseases, emerging pathogens, and rapidly evolving microbes. Due to globalization and increased travel, emigration, and migration, biosurveillance is critical throughout the world, not just in pockets of more developed regions.

Building up the capabilities and capacities for biosurveillance is a global challenge. Cooperative biological engagements help address biosurveillance and biosafety gaps in capabilities and reduce threats worldwide, by strengthening biosurveillance globally in a number of ways. The first is in assisting countries and regions to increase their technical expertise for detecting, diagnosing, and reporting on rapidly changing and emerging infectious diseases. Second, cooperation can help strengthen the biosafety and biosecurity of laboratories around the world. Third, biosurveillance can be strengthened by understanding the best strategies for biosurveillance planning, and the potential epidemiology of a disease system within a region. In these instances, collaborative research comes into play to help scientists understand a disease system in the environment and devise the most effective strategy for detecting outbreaks. The articles in the Frontier Topic “Biological Engagement Programs: Reducing Threats and Strengthening Global Health Security Through Scientific Collaboration” cover each of these primary areas of international collaboration. This topic brings together 148 authors from over 25 countries with the shared mission of reducing the threat of infectious diseases.

Reducing the threat of a nefarious use of pathogens on any human or animal population is a top priority for global security. Specifically, the Global Health Security Agenda (GHSA) is “an effort by nations, international organizations, and civil society to accelerate progress toward a world safe and secure from infectious disease threats; to promote global health security as an international priority; and to spur progress towards reducing infectious diseases” (1). Working with partner countries around the world, the GHSA will be focused on mitigating the impact of naturally occurring outbreaks and intentional or accidental releases of dangerous pathogens; assisting countries to rapidly detect and transparently report outbreaks when they occur; and employing an interconnected global network that can respond rapidly and effectively. The most important component of the GHSA is international cooperation (Galloway et al.). Galloway et al. present a review of the GHSA and the importance of being proactive in the new era of globalization. In addition, Standley et al. give a history of Cooperative Threat Reduction programs and how cooperative bioengagements can assist in the implementation of the International Health Regulations.

Cooperative engagements and collaborations across borders help foster open communication and sharing of data. Being aware of the transboundary nature of pathogens helps break down the barriers to sharing information between countries, and cooperative engagement programs are designed to build a foundation of trust that can help lessen potential negative aspects of sharing data such as economic or political consequences. The fight against infectious diseases is shared by humanity; reducing individual infections and outbreaks of zoonotic diseases in humans, agricultural animals, and wildlife is a shared goal across the world. Fair et al. present a model for measuring the return on relationships of collaborations and the resulting networks of people that remain in place after trainings or projects are complete.

## Biosafety and Biosecurity Challenges

Sampling and laboratory analysis for infectious diseases requires a certain amount of infrastructure and unique skills in molecular techniques in virology and bacteriology. Samples may have to be cultured and saved for future reference, and the microbiology environment for working with such pathogens must be both safe and secure. Best practices for biosafety and biosecurity are often learned through previous mistakes in the field and laboratory. Sharing these lessons learned is a critical factor in strengthening the biosafety and biosecurity environment in laboratories around the world. Khan et al. discuss biosafety initiatives and gaps in the BMENA region. In addition, Al Jewari and Koblentz share how to strengthen biosecurity in Iraq and the development of an Iraqi National Biorisk Management System.

## “One World, One Health” Unification

The One World, One Health (OWOH) agenda is based on the foundation that most pathogens continually circulate in animal species and that there is a constant interplay between agricultural animals, wildlife, the environment, and humans. Therefore, the OWOH agenda is focused on surveillance, biosecurity, and biodiversity developed too limit infectious agents in a synergistic manner with animals, humans, and the environment. The unified and holistic approach to OWOH health was established in 2004 at a New York meeting where 12 principles were defined for multidisciplinary and integrated approaches to health. Over the last decade, the One Health approach has been applied to disease situations around the world and while some sociological challenges have been identified (2, 3), many success stories can also be told. As a common outcome of biological cooperative engagement projects, Ministries of Health in over 25 countries worldwide have worked closely with Ministries of Agricultural, leading to more communication, sharing, and cooperation on zoonotic diseases across disciplines. Several papers in this Frontiers Topic review efforts to increase capabilities for biosurveillance such as developing genomic capabilities for detecting pathogens by Cui et al.

## How Can Research Help Address the Challenge of Outbreaks?

Many of the papers in this Frontiers Topic highlight collaborative research on infectious pathogens of security concern. For example, Bartholomew et al. review the history of building infectious disease research programs with countries of the Former Soviet Union. Scientific research on infectious diseases often focuses on reductionism, or understanding the molecular and physiological mechanisms of host–pathogen interactions. Research may also focus on a higher scale of understanding the disease “system.” Several papers in this collection highlight studies for understanding the diseases systems, such as Kokashvili et al. reporting on Vibrio species in the aquatic environment of Georgia, and the epizootology of Lumpy Skin disease in livestock in Azerbaijan by Zeynalova et al.

Cooperative biological engagement research tends to focus on the higher system-level scale since its objective is to increase the effectiveness of biosurveillance. For example, understanding a disease “system” such as Middle East Respiratory Syndrome coronavirus in the Middle East can lead to insights into the transmission events as well as better detection and possible mitigations to stop the infectious cycle. Understanding a disease system may sometimes require gathering information that may appear irrelevant to the disease, but may be critical for comprehending its spread. For example, mapping the distribution of bat species in a region and their migratory patterns can provide vital clues as to how and why disease outbreaks keep occurring or are emerging. Host range and host heterogeneity are important aspects of a disease system, as is identifying dead end hosts, regular host, and potential “super-spreaders.”

With limited monetary resources for biosurveillance, efforts need to be as directed and thoughtful as possible in order to be cost effective and successful. Developing the best strategy for biosurveillance requires knowledge of disease systems and that requires methodical and hypothesis-based scientific research. The last and most critical step is then applying the knowledge learned from scientific studies to inform policies. Blackburn et al. share examples of the applications of research on infectious diseases to policies for mitigating and responding to disease outbreaks. Two other papers by Horn and Hay et al. discuss the challenges of doing cooperative research in austere environments with take-home lessons for all future cooperative science engagements [Hay et al.; Horn].

## Global Challenges Require Global Collaboration

Rates of evolution of phenotypic traits in species vary widely in a continuum of slow to rapid evolution. Species may adapt to environmental changes differently and in the instance of climate change, species that are not able to adapt to a rapidly changing environment may be worse off than species that can. Clear evidence is mounting that changes in mean temperature or climate variability are increasing infectious disease risk globally (4, 5). Cooperation will continue to be important as vectors, hosts, and pathogens shift their ranges and seasonality.

Selection pressures may also force rapid evolution in species with short generation times, such as microbes. Antimicrobial resistance (AMR) is an example of rapid evolution in response to selection pressures, primarily in response to antimicrobial drugs. AMR is now considered a major global threat to public health (6, 7). In 2015, the World Health Assembly endorsed a global action plan to tackle AMR, with a primary focus on antibiotic resistance (8). AMR is occurring everywhere in the world, compromising the ability to treat infectious diseases with life-saving drugs of the past such as penicillin. The goal of the global action plan is to ensure, for as long as possible, continuity of successful treatment and prevention of infectious diseases with effective and safe medicines that are quality-assured, used in a responsible way, and accessible to all who need them. Again, because antimicrobial selection pressures may vary between country and region, international collaboration is required to tackle the challenge of increasing antimicrobial-resistant pathogens. Antimicrobial-resistant microbes also know no borders.

The scientists and authors who have come together in this Frontiers Topic on cooperative biological engagements have a shared passion and mission for both reducing the threat of infectious diseases, and international collaboration and coordination. Coming together across the globe allows for a greater diversity of ideas that then leads to more innovation and creative problem solving. Shared insights from direct experiences and research increase the ability to reduce infectious disease outbreaks. Reducing outbreaks, epidemics, and pandemics potentially saves thousands of lives. While it has always been difficult to “prove a negative” for the effectiveness of programs such as cooperative biological engagements, the success stories are there and the scientific research that comes from such programs is invaluable. We are indebted to the work of everyone involved in such programs around the world, and especially to the authors contributing to this special Frontiers Topic.

## Author Contributions

This is a single author paper by the primary Editor for the Topic “Biological Engagement Programs: Reducing Threats and Strengthening Global Health Security Through Scientific Collaboration.” This editorial that introduces the Topic was completely written by JF.

## Conflict of Interest Statement

The author declares that the research was conducted in the absence of any commercial or financial relationships that could be construed as a potential conflict of interest.
